# Inflammation and epithelial–mesenchymal transition in a CFTR‐depleted human bronchial epithelial cell line revealed by proteomics and human organ‐on‐a‐chip

**DOI:** 10.1111/febs.70050

**Published:** 2025-03-03

**Authors:** Domenico Mattoscio, Luis A. Baeza, Haiqing Bai, Tommaso Colangelo, Simone Castagnozzi, Marta Marzotto, Maria Concetta Cufaro, Virginia Lotti, Yu‐Chieh Yuan, Matteo Mucci, Longlong Si, Mariachiara Zuccarini, Maria Tredicine, Simona D'Orazio, Damiana Pieragostino, Piero Del Boccio, Claudio Sorio, Marco Trerotola, Mario Romano, Roberto Plebani

**Affiliations:** ^1^ Department of Medical, Oral and Biotechnological Sciences “G. d'Annunzio” University Chieti‐Pescara Italy; ^2^ Center for Advanced Studies and Technology (CAST) “G. d'Annunzio” University Chieti‐Pescara Italy; ^3^ Xellar Biosystems Boston MA USA; ^4^ Department of Medical and Surgical Sciences University of Foggia Italy; ^5^ Unit of Cancer Cell Signalling IRCCS Casa Sollievo della Sofferenza San Giovanni Rotondo (FG) Italy; ^6^ Department of Medicine, Division of General Pathology University of Verona Italy; ^7^ Department of Innovative Technologies in Medicine and Dentistry “G. d'Annunzio” University of Chieti‐Pescara Chieti Italy; ^8^ Section of Microbiology, Department of Diagnostics and Public Health University of Verona Italy; ^9^ CAS Key Laboratory of Quantitative Engineering Biology Shenzhen Institute of Synthetic Biology, Shenzhen Institute of Advanced Technology Chinese Academy of Sciences Shenzhen China; ^10^ Department of Science University “G. d'Annunzio” of Chieti‐Pescara Chieti Italy

**Keywords:** CRISPR/Cas9, cystic fibrosis, epithelial–mesenchymal transition, organ‐on‐a‐chip, proteomics

## Abstract

Cystic fibrosis (CF) is a genetic disease caused by mutations in the CF transmembrane conductance regulator (*CFTR*) gene, leading to chronic, unresolved inflammation of the airways due to uncontrolled recruitment of polymorphonuclear leukocytes (PMNs). Evidence indicates that CFTR loss‐of‐function, in addition to promoting a pro‐inflammatory phenotype, is associated with an increased risk of developing cancer, suggesting that CFTR can exert tumor‐suppressor functions. Three‐dimensional (3D) *in vitro* culture models, such as the CF lung airway‐on‐a‐chip, can be suitable for studying PMN recruitment, as well as events of cancerogenesis, that is epithelial cell invasion and migration, in CF. To gather insight into the pathobiology of CFTR loss‐of‐function, we generated CFTR‐knockout (KO) clones of the 16HBE14o‐ human bronchial cell line by CRISPR/Cas9 gene editing, and performed a comparative proteomic analysis of these clones with their wild‐type (WT) counterparts. Systematic signaling pathway analysis of CFTR‐KO clones revealed modulation of inflammation, PMN recruitment, epithelial cell migration, and epithelial–mesenchymal transition. Using a latest‐generation organ‐on‐a‐chip microfluidic platform, we confirmed that CFTR‐KO enhanced PMN recruitment and epithelial cell invasion of the endothelial layer. Thus, a dysfunctional CFTR affects multiple pathways in the airway epithelium that ultimately contribute to sustained inflammation and cancerogenesis in CF.

## Graphical abstract

Proteomic analysis of cystic fibrosis transmembrane conductance regulator knockout (CFTR‐KO) clones of 16HBE14o‐ cells showed altered pathways linked to inflammation, polymorphonuclear leukocyte (neutrophil) recruitment, and epithelial–mesenchymal transition. These findings were validated by cytokine analysis and functional experiments exploiting the organ‐on‐a‐chip technology.

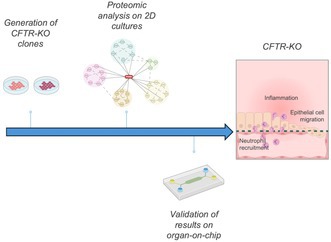

**Abbreviations**
CF: cystic fibrosis
CFTR: cystic fibrosis transmembrane conductance regulator
EMT: epithelial‐to‐mesenchymal transition
LUAD: lung cancer data set
NSCLC: non‐small cell lung cancer
PMN: polymorphonuclear leukocytes
VEGF: vascular endothelial growth factor

## Introduction

Cystic fibrosis (CF) is a genetic disease caused by mutations in the CF transmembrane conductance regulator (*CFTR*) gene on chromosome 7 [1]. The CFTR protein directly regulates the flux of chloride and bicarbonate ions across the cell membrane and, indirectly, the transport of sodium by acting on the epithelial sodium channel [2]. In the lung, CFTR loss‐of‐function causes contraction of the periciliary volume, accumulation of dense mucus, chronic infection, and neutrophilic inflammation, which lead to respiratory insufficiency and death [3].

Unresolved inflammation is a trademark of CF lung disease [4] and evidence indicates that CFTR loss‐of‐function promotes a constitutive pro‐inflammatory phenotype, even in the absence of infection [5]. Notably, the CFTR protein is expressed in cells from varying tissues, including the pancreas [6], liver [7], intestine [8], as well as in cells involved in the immune response, such as endothelial cells [9, 10, 11], macrophages [12], monocytes [13], and platelets [14, 15]. Thus, although lung disease has predominant clinical relevance, CF should be considered a systemic, multi‐organ disease.

Epithelial‐to‐mesenchymal transition (EMT) has been observed in CF [16], suggesting a correlation between CFTR loss‐of‐function and malignancy [17]. Consistent with this, CF was found to be associated with up to 20‐fold increased risk of developing digestive tract cancers [18, 19, 20, 21, 22]. Moreover, CFTR was reported to be hypermethylated and downregulated in a number of metastatic cancers, including non‐small cell lung cancer (NSCLC) [23]. In this context, CFTR was identified as an inhibitor of NSCLC metastasis and a potentially novel biomarker of malignant progression in NSCLC patients [24]. Thus, the role of CFTR as a regulator of cancer development and progression is emerging [25].

The organ‐on‐a‐chip technology has revolutionized preclinical research, becoming a tool with unprecedented reliability for studying multiple aspects of the immune‐inflammatory response, including the recruitment of immune cells during infections by respiratory viruses or bacteria [26, 27, 28]. We recently established the first CF airway‐on‐a‐chip model to investigate inflammation and pathogen infection in the CF airways [29]. This model, composed of epithelial and endothelial cells, may also represent a suitable tool for assessing epithelial cell invasion and migration, as well as an EMT phenotype.

In the present study, we generated three CFTR‐KO clones of the human airway epithelial cell line, 16HBE14o‐, using CRISPR/Cas9 gene editing. We consistently observed that, compared with the WT counterpart, these clones displayed upregulation of proteins involved in inflammation, PMN recruitment, and EMT‐related migration. Moreover, when cultured under flow in microfluidic chips, the CFTR‐KO cells were associated with enhanced PMN recruitment and epithelial cell migration.

These results support the view that, in addition to the regulation of the immune‐inflammatory response, CFTR may play roles in EMT and malignant progression.

## Results

### CFTR‐KO in human respiratory epithelial cells using the CRISPR/Cas9 system

**Fig. 1:**
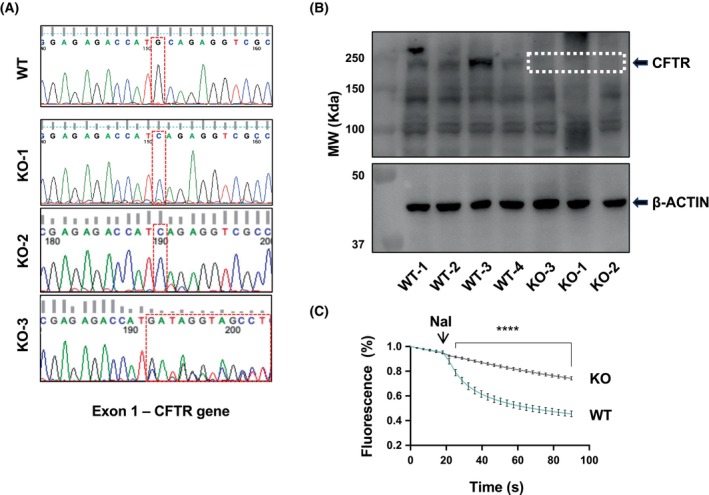
Cystic fibrosis transmembrane conductance regulator (CFTR) ablation by CRISPR/Cas9 mutagenesis. (A) DNA sequencing of single guide RNA (sgRNA) targeting region in one wild‐type (WT) (upper image) and three CFTR‐knockout (KO) 16HBE14o‐cells (lower image). The electropherograms refer to the region of exon 1 targeted by the CRISPR/Cas9 system. The red box in the control clone (top) refers to the ‘G’ corresponding to the ATG of the first codon, whereas in the KO clones (bottom) is referred to the changes in the WT sequence. (B) Western blot analysis of CFTR expression using 596 mouse anti‐human CFTR antibody (UNC, see Methods for details) diluted 1:1000 with 3% milk in TBS‐T (Top). The lower part of the membrane was incubated with the anti β‐Actin antibody (#A5441; Sigma‐Aldrich™) diluted 1:5000 with 3% milk in TBS‐T (Bottom). The white box refers to the portion of the membrane corresponding to the C band of CFTR protein, which was not detected in KO clones. The western blot shown is representative of *n* = 2 experiments. (C) CFTR activity assay by yellow fluorescent protein (YFP) (see Methods for details) in WT and KO‐1 clones. 90‐s videos were recorded to assess the fluorescence decay after the addition of sodium iodide (NaI). Data points are mean ± SEM from six replicates of one experiment, each containing the fluorescence intensity of 10 cells. ****P < 0.0001 from 25 to 90 s (two‐way ANOVA and Sidak's multiple‐comparison test).

To generate CFTR‐knockout (KO) cell lines, we infected 16HBE14o‐ cells with CRISPR/Cas9 viral particles containing *CFTR*‐targeting sgRNA or a negative sgRNA control. After viral infection and single‐cell clone generation, PCR amplification, and sequencing of the targeted genomic DNA region showed successful changes across the CFTR translation start site (TSS) in exon 1. Specifically, we found a single guanine deletion in both alleles of the translation start site ATG in two KO clones (KO‐1 and KO‐2), with consequent frameshift mutations at the very beginning of the CFTR mRNA. An additional clone (KO‐3) showed the same mutation as KO‐1 and KO‐2 in one of the two CFTR alleles and a different mutation in the second allele (Fig. 1A). One random negative control clone displayed no changes in the CFTR sequence (Fig. 1A). Western blot analysis showed a complete depletion of the CFTR protein in the KO clones, consistent with a premature termination of translation (Fig. 1B). We also measured CFTR anion‐transport activity using a live fluorescence microscopy assay based on the YFP‐H148Q halide sensor, as reported by Galietta *et al*. [30]. Consistent with the western blot results, we did not detect CFTR activity in KO cells (Fig. 1C).

### Proteomic analysis of CFTR‐KO cells

**Fig. 2:**
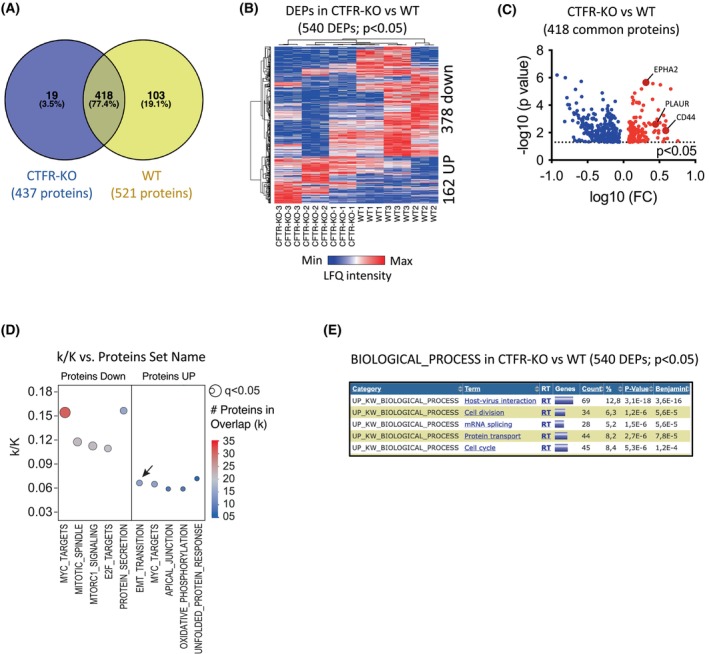
Comparative proteomic analysis between cystic fibrosis transmembrane conductance regulator (CFTR) wild‐type (WT) and knockout (KO) clones. (A) Venn diagram representing the overlap of expressed proteins in WT (*n* = 521) and CFTR‐KO (*n* = 437) cells. (B) Heat map of significant differentially expressed proteins (DEPs) in CFTR‐KO vs. WT cells (*P*‐value <0.05; Student *t*‐test). (C) Volcano plot of common DEPs in CFTR‐KO vs. WT cells. The color intensity indicates the level of protein expression: red, upregulated, and blue, downregulated. FC = fold change. *P*‐value was calculated using the Student's *t*‐test. (D) MSigDB‐overlap analysis of DEPs as in (B) with the Hallmark (H) gene sets (*N* = 50). The bubble plot shows the top five overlapping protein sets among CFTR‐KO‐regulated proteins (these sets include the EMT transition—arrow). Bubble size represents the statistical significance of overlap expressed as −log_10_(*q*‐value), where the larger the size, the greater the significance. In the x‐axis, H‐gene set name; in y‐axes, the ratio of overlap (k/K) is shown, where ‘k’ represents the number of CFTR‐KO regulated proteins while ‘K’ is the number of genes in the specific H‐protein set. Bubble color reflects the number of CFTR‐KO DEPs (k). The complete results are provided in Tables S3 and S4. (E) Analysis of Biological Processes activated in CFTR‐KO vs. WT cells (540 DEPs; *P*‐value < 0.05) using UniprotKB Keywords (UP KW) in DAVID. Each bar represents a distinct biological process, with the length of the bar indicating the number of proteins associated with that process. The analysis shown in this figure was created using the values in the protein list in Table S1 (triplicates of *n* = 3 for both WT and KO clones).

To uncover pathways regulated by CFTR in airway cells, we carried out proteomic analysis of CFTR‐WT and CFTR‐KO clones. To this end, we analyzed protein lysates from 3 WT and 3 KO clones in triplicate using mass spectrometry. We identified a total number of 1859 proteins in the CFTR‐KO lysates and 2009 proteins in the WT controls (Table S1). We found that 540 proteins were differentially expressed between the KO and WT clones; out of them, 19 proteins were exclusively detected in the CFTR‐KO clones, and 103 were exclusively expressed by the WT controls (Fig. 2A). Overall, the KO clones showed 378 downregulated proteins and 162 upregulated proteins compared with their WT counterpart (Fig. 2B; Tables [Link], [Link], [Link]). Of note, the KO clones showed marked overexpression of CD44, PLAUR, and EPHA2 (Fig. 2C), pivotal EMT modulators [31, 32, 33]. Molecular Signature Database (MsigDB) analysis (see Methods) of the 540 differentially expressed proteins (DEPs) underlined a significant association of the CFTR gene with relevant cancer‐related pathways. In detail, the 378 downregulated proteins in KO cells revealed an enrichment in the top significant signatures with the lowest *P*‐values, including targets of the c‐Myc oncogene, the mammalian target of rapamycin gene complex 1 (mTORC1), and the E2F family of transcription factors. On the contrary, the 162 upregulated proteins in KO cells showed a significant representation of the top‐ranked ‘EMT program’, as well as c‐Myc targets and the apical junction pathway. Several cancer‐related signaling pathways were modified upon inhibition of CFTR, such as those regulating cell cycle and division, mitotic spindle, protein secretion, EMT, apical junctions, oxidative phosphorylation, and endoplasmic reticulum response to error‐induced stress (Fig. 2D,E; Tables S3 and S4). Furthermore, consistent with the role of CFTR in the lung parenchyma [34], we observed modulation of proteins that characterize host‐virus interaction and protein transport (Fig. 2E, Table S5).

**Fig. 3:**
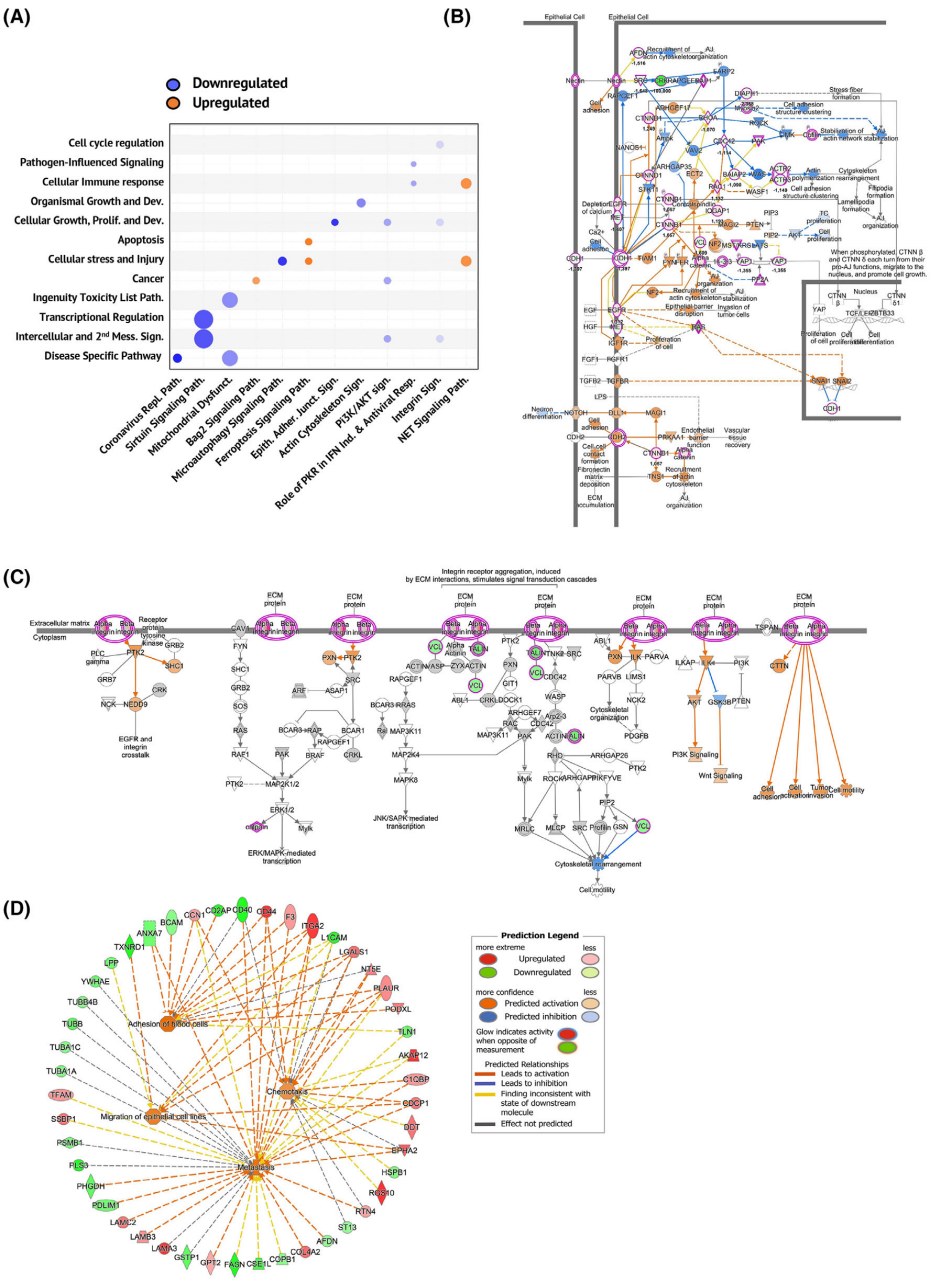
Ingenuity Pathway Analysis (IPA) of proteomic data. Cystic fibrosis transmembrane conductance regulator (CFTR)‐knockout (KO) epithelial clones showed a proteomic profile indicative of increased immune cell recruitment and spreading. The analysis was performed using the values in the protein list in Table S1 (*n* = 3 for both WT and KO clones). (A) Bubble plot of top canonical pathways predicted by IPA with z‐score value, which represents a measure of the predicted direction of the pathway activity. Each bubble represents a canonical pathway, and the bubble size is directly proportional to the number of proteins that overlap the pathway. Bubble color represents z‐scores as per the legend. (B) IPA representation of proteins involved in epithelial junctions. (C) IPA representation of proteins involved in integrin receptor signaling. (D) Left: Graphical summary of the major biological themes in KO clones as determined by IPA core analysis of DEPs. Right: legend relative to panels B–D. Red proteins: upregulated in KO clones; green proteins: downregulated in KO clones, with the intensity of colored infill indicating the level of up or downregulation, respectively. The purple outline indicates differentially expressed molecules in our dataset. Predicted modulated proteins and functions in KO clones are represented in blue (downregulated) or orange (upregulated). Yellow lines indicate nonconsistent relationships. Lines indicate relationships leading to activation (orange) or inhibition (blue). Gray lines point to nonpredicted effects. Solid lines represent direct interactions, whereas dashed lines represent indirect interactions.

Orthogonal analysis of the proteomic data using Ingenuity Pathway Analysis (IPA) revealed alterations of several processes in the CFTR‐KO clones, including cell cycle regulation, cellular immune response, organismal growth and development, cellular growth, proliferation and development, apoptosis, viral infection, immune cell recruitment, and cancer (Fig. 3A). Furthermore, protein interaction analysis predicted a significant modulation, among others, of coronavirus replication, sirtuin signaling, epithelial adherent junctions, and Neutrophil Extracellular Trap (NET) signaling (Fig. 3A). Of note, the pathway involved in host‐virus interaction is of interest as we and others recently reported a different susceptibility to SARS‐CoV‐2 infection/replication in CFTR defective cells in comparison with normal ones [35]. IPA analysis of CFTR‐KO cells vs. WT controls also revealed inhibition of E‐cadherin (CDH1)‐dependent adhesions (Fig. 3B), which represent a crucial step in EMT [36], ablation and stimulation of integrin‐dependent cell migration (Fig. 3C), metastatic spreading, chemotaxis, and adhesion of immune cells, as reported in the functional network (Fig. 3D).

### The CFTR‐KO cells exhibit a pro‐inflammatory secretome and increased PMN recruitment

**Fig. 4:**
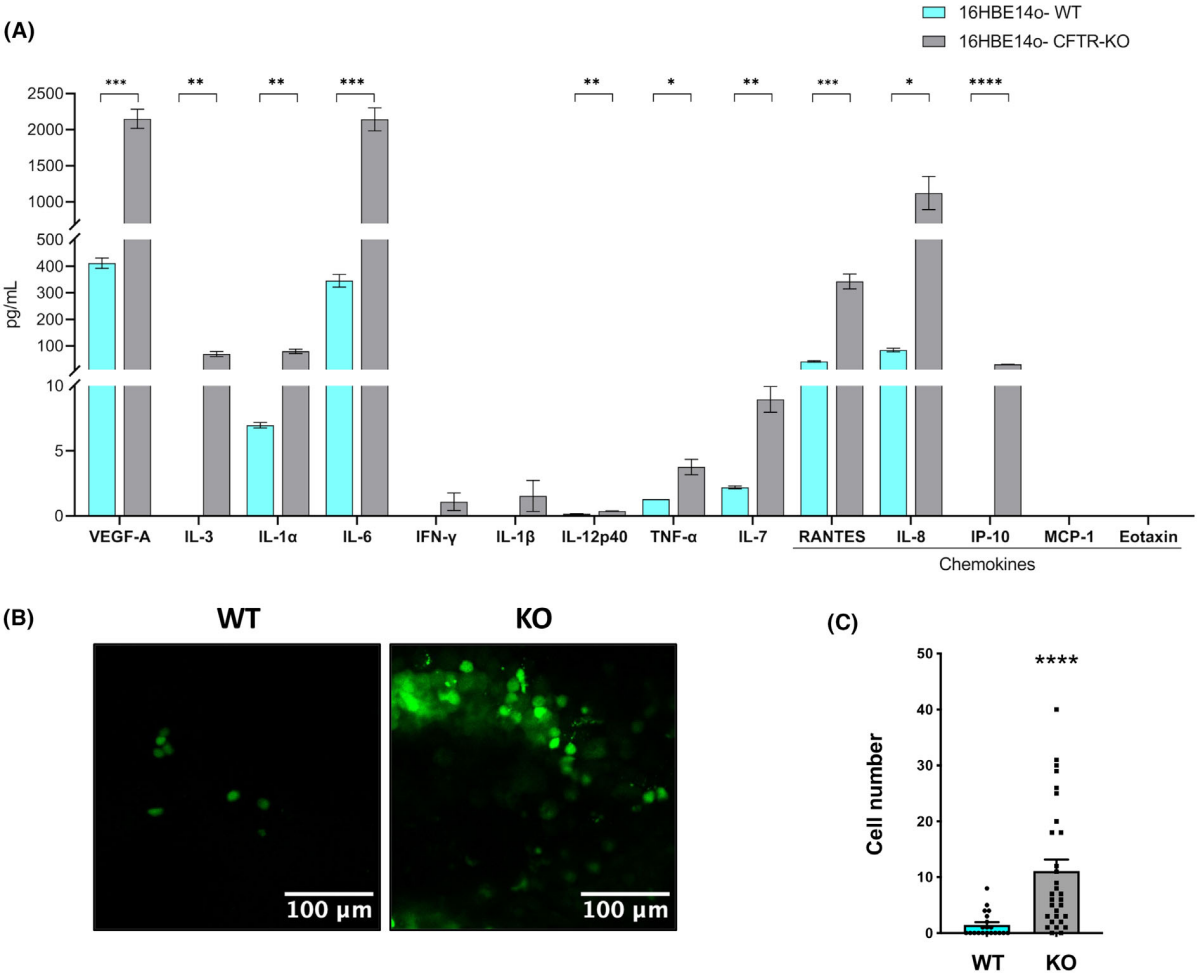
Cystic fibrosis transmembrane conductance regulator (CFTR)‐knockout (KO) related inflammatory profile. (A) Cytokine and chemokine released by resting wild‐type (WT) and CFTR‐KO 16HBE14o‐ in 24 h. Bars represent mean ± SD of *n* = 3. *P*‐values were adjusted with the Bonferroni‐Dunn multiplicity test: *P < 0.05, **P < 0.01; ***P < 0.001, ****P < 0.0001. (B) Representative immunofluorescence images (one out of 20 for CFTR‐WT and one out of 30 for CFTR‐KO) of CFSE‐live‐stained polymorphonuclear PMN in the lower membrane side of WT‐(left) and CFTR‐KO (right) chips. Scale bars: 100 μm. (C) PMN cell number quantification in WT and KO chips. Dots represent the PMN count within 10 random images per each chip acquired along the entire path of the channel (20 dots from *n* = 2 WT chips and 30 dots from *n* = 3 KO chips from one experiment). Mean ± SEM is also shown. Nonparametric data distribution was assessed using the Mann–Whitney T‐test ****P < 0.0001.

To further analyze the pro‐inflammatory signaling induced by inhibition of CFTR, we quantified the levels of a panel of cytokines and chemokines secreted by the WT and CFTR‐KO cells in the culture medium. As shown in Fig. 4A, the levels of released pro‐inflammatory cytokines were significantly higher in supernatants from CFTR‐KO compared with WT cells. In particular, IP‐10, VEGF‐A, RANTES, and IL‐6 levels exhibited a strong, statistically significant difference between CFTR‐KO and WT cells. The increase of these pro‐inflammatory cytokines in CFTR‐KO cells supported a corresponding modulation of PMN recruitment, a major pathogenetic event occurring in CF lung disease [37]. To investigate the dynamic recruitment of PMN to an epithelial layer of WT or CFTR‐KO cells, we employed an organ‐on‐a‐chip microfluidic device to co‐culture WT or KO cells with pulmonary artery endothelial cells (PAEC) for 3 days under continuous flow. We then perfused CFSE‐live‐stained PMN in the vascular channel of the chips for 30 min and evaluated their recruitment to the interface between the vascular and the epithelial channel using confocal microscopy. We consistently found that an increased number of PMN adhered to the porous membranes at the channel interface in the CFTR‐KO chips compared with the WT chips (Fig. 4B,C). This is consistent with the increased immune cell recruitment predicted by the IPA analysis (Fig. 3A,D).

### Loss of CFTR correlates with a more invasive phenotype

**Fig. 5:**
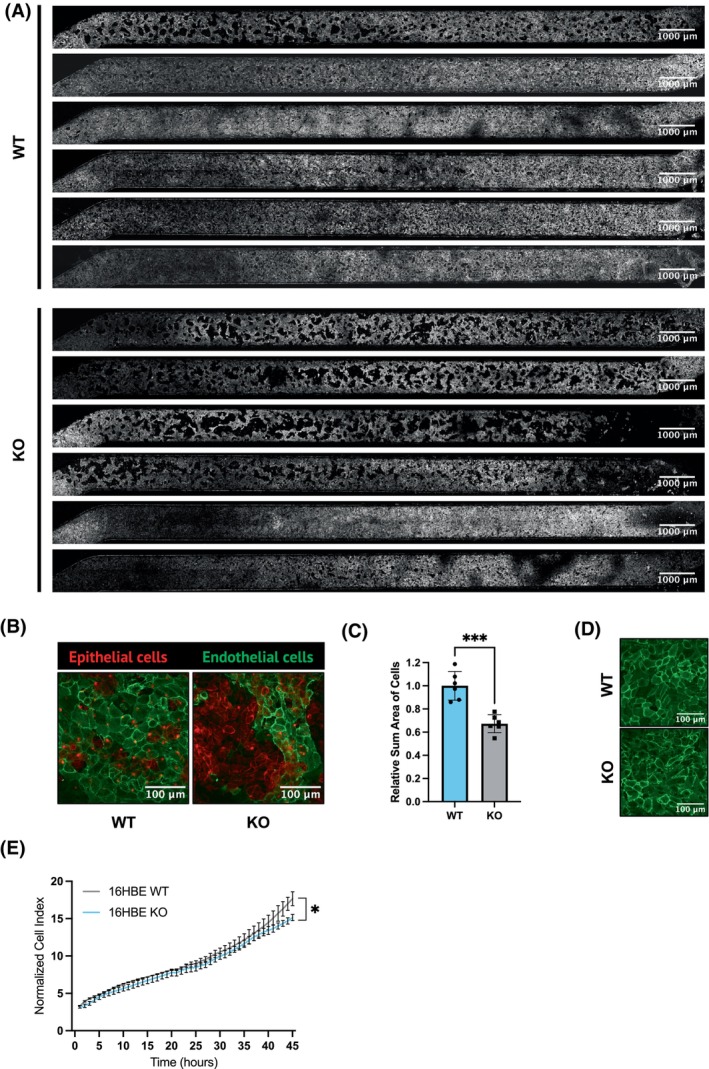
Invasive phenotype of cystic fibrosis transmembrane conductance regulator (CFTR)‐knockout (KO) cells. (A) Tile imaging of endothelial cell (EC) distribution in the lower surface of the membrane that divides the two channels of *n* = 6 WT + 6 KO chips. Processed images after noise reduction are shown. Scale bars: 1000 μm. (B) Confocal images showing EPCAM‐stained epithelial cells (red) infiltrating CD31‐stained EC (green) in the lower surface of the membrane. Scale bars: 100 μm. Representative images of *n* = 6 WT and 6 KO chips. (C) Quantification of the relative sum area occupied by ECs in the lower side of the membrane of *n* = 6 WT and 6 KO chips shown in panel A. Bars represent mean ± SD of a total number of *n* = 6 for WT and *n* = 6 for KO chips derived from two experiments. Data were analyzed using an unpaired T‐test. ***P < 0.001. (D) Integrity of the endothelial layer in the lower side of the bottom channel. Scale bars: 100 μm. (E) Relative growth of WT or KO 16HBE14o‐analyzed with impedance‐based real‐time cell analysis (ACEA). Data are expressed as relative cell growth normalized at the time of cell seeding on the plate. Data points are mean ± SEM from 2 technical replicates. *P < 0.05 at 45 h (two‐way ANOVA and Sidak's multiple‐comparison test).

To strengthen the proteomic prediction of the impact of CFTR‐KO on cell adhesion and EMT activation, we exploited the organ‐on‐a‐chip technology. To this end, we cultured, immuno‐stained, and imaged by confocal microscopy airway chips populated by WT or CFTR‐KO clones. We observed extensive damage to the endothelial monolayer on the lower side of the membrane at the interface between the two channels (Fig. 5A). Moreover, co‐staining with antibodies against EpCAM (epithelial marker) and CD31 (endothelial marker) revealed the presence of epithelial cells in the endothelial cell channel adjacent to the membrane, both in chips populated by KO clones and those populated by WT cells (Fig. 5B). However, image quantification of the whole basal channels showed that the area occupied by endothelial cells in the CFTR‐KO chips was significantly smaller compared with that in the WT chips (Fig. 5C). This suggests that CFTR loss‐of‐function increases lung epithelial cell invasiveness. We verified that the endothelial cells lined the lower side of the vascular channel as a compact monolayer, thus ruling out the possibility that the discontinuity of the endothelial monolayer adjacent to the porous membrane might have been caused by some technical artifacts, such as uneven flow or the presence of air bubbles (Fig. 5D). In addition, during EMT, cells often display alterations of their proteome that affect their ability to proliferate, with some transformed cells showing increased proliferation [38] and others displaying decreased proliferation [39]. This is dependent on whether the cells use their energy to proliferate or increase their motility and attempt to invade [39]. In this context, our IPA analysis revealed a reduction in pathways related to growth, proliferation, and development (Fig. 3A). To further investigate this aspect, we performed a real‐time impedance growth curve assay. Notably, we observed a slight but significant decrease in the growth rate of CFTR‐KO cells compared with WT control cells (Fig. 5E). These findings correlate with and support the invasiveness feature with aspects of EMT described in lung cancer research [40].

**Fig. 6:**
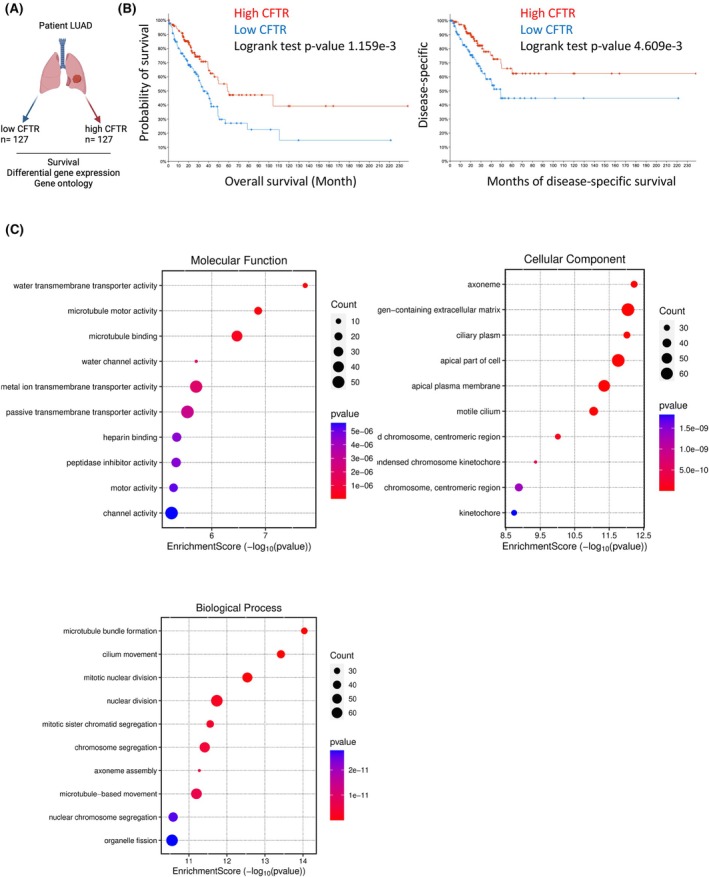
Low cystic fibrosis transmembrane conductance regulator (*CFTR*) expression drives cancer hallmarks in lung adenocarcinoma (LUAD). (A) Diagram of the experimental strategy and analyses. (B) Kaplan–Meier curve survival analysis (overall and disease‐free survival) of LUAD patients stratified for high (*n* = 127) or low (*n* = 127) *CFTR* expression score. Log‐rank test *P*‐value is reported. (C) Gene Ontology (GO) of enriched molecular functions, cellular components, and biological processes in high vs. low *CFTR* tumors as identified by SRplot. GO terms, gene count, enrichment score, and *P*‐values.

To confirm whether CFTR could also modulate oncogenic potential *in vivo*, we leveraged the Cancer Genome Atlas (TCGA) lung cancer data set [lung adenocarcinoma (LUAD)]. Using RNA‐seq datasets integrated with clinical characteristics, we stratified tumors into low vs. high *CFTR* LUAD according to their *CFTR* expression as a proxy for CFTR impact on lung cancer (Fig. 6A). Survival analysis in these subgroups revealed that patients with lower *CFTR* expression had worse outcomes in terms of overall and disease‐free survival (Fig. 6B), confirming previous reports suggesting a role for CFTR as a tumor suppressor also in lung cancer [24, 41, 42]. To gain further insights on how CFTR could promote lung cancer progression, we performed a Gene Ontology (GO) analysis of the DEG among low vs. high *CFTR* tumors. Enrichment analysis highlighted some expected molecular functions associated with CFTR, channel activity, ion and water transport, and apical membrane as cellular components, thus validating our approach (Fig. 6C). However, we also identified key molecular and cellular hubs related to key cancer hallmarks, such as cytoskeletal dynamics, that are involved in EMT and cell migration [43] and cellular replication. Taken together, these results suggest that in human lung cancer, the loss of CFTR function may disrupt cytoskeletal organization and trigger the acquisition of the EMT phenotype that drives cancer progression.

## Discussion

With the extension of life expectancy for people with CF, the question of whether CFTR loss‐of‐function may have an impact on pathogenetic mechanisms of aging‐related disorders, including cancer, becomes relevant. To study these aspects, we utilized the CRISPR/Cas9 technology to generate CFTR‐KO human respiratory cell clones (Fig. 1), which we subjected to proteomic analysis and functional validation with a human‐relevant airway chip model. Primary cells cultured at an air‐liquid interface can accurately mimic human airways, faithfully reproducing features, such as polarization, barrier function, and tight junctions [44]. However, their limited culture propagation makes them unsuitable for clonal generation. Recognizing the trade‐off of foregoing some key characteristics of primary cells, we chose the 16HBE14o‐ cell line, which has been extensively used in CF research. This cell line expresses high levels of *CFTR* mRNA and protein, and it is therefore a suitable model to study the molecular mechanisms [45, 46, 47] and consequences of CFTR depletion by genetic manipulation. Importantly, 16HBE14o‐ cells form tight junctions and exhibit robust barrier properties during differentiation, both in submerged cultures [48] and at the air‐liquid interface [49], making our generated clones also suitable for studying epithelial properties that critically influence a variety of functions, including EMT [50]. Analyses of proteomic data consistently revealed that loss of CFTR was associated with the dysregulation of pathways and functions related to cancer (EMT, cell adhesion and migration, chemotaxis, metastatic spreading, and cell division), as well as to the host response (NET signaling, immune cell recruitment, host‐virus interactions) (Figs 2 and 3). This evidence is consistent with the view that CFTR is a multifunctional protein, in line with its complex intracellular interactome [51, 52]. It should be noted that despite bronchial cells expressing CFTR protein, this is not listed in the proteomic results. This is not unexpected since some hydrophobic features of membrane proteins make their detection with standard LC–MS/MS difficult, often requiring enrichment strategies and specific lysis buffers.

The involvement of CFTR in cancer development and progression has been documented by several experimental and clinical studies [25]. Correlations between CFTR dysregulation and invasiveness, therapeutic response, and prognosis have been reported in different types of cancer. For instance, people carrying *CFTR* mutations presented a higher risk of developing colorectal cancer than individuals without *CFTR* mutations [53]. Moreover, CFTR was downregulated in human breast cancer in association with poor prognosis [17]. CFTR downregulation was also observed in nasopharyngeal carcinoma (NPC), with inhibition of NPC cell migration and invasion by overexpression of CFTR and an increment in these activities by CFTR‐KO [54]. In addition, low CFTR expression caused by hypermethylation of the gene promoter has been associated with poor survival in young lung cancer patients [24], also confirmed in the present report.

Notably, our proteomic data showed that CFTR‐KO was associated with the downregulation of the sirtuin signaling pathway (Fig. 3). This may be relevant in cancer biology, given the regulatory actions of sirtuins on multiple cancer‐related processes and the evidence of the tumor‐suppressive function of some sirtuins in several neoplasms, including breast, lung, and colon cancer [55]. Likewise, the upregulation of the BAG‐2 pathway by CFTR‐KO (Fig. 3) is consistent with the tumor‐suppressive actions of CFTR, in consideration of the involvement of BAG‐2 in cancer growth and spread [56].

Along these lines, CFTR‐KO led to markedly enhanced expression of CD44, PLAUR, and EPHA2, key components of the EMT pathway (Fig. 2). This is consistent with the evidence that mutant CFTR cells and tissues exhibited signs and biomarkers of EMT [57, 58], as well as with the evidence that, in colorectal cancer, CFTR blockade upregulated EMT markers [59]. EMT involves the acquisition of a mesenchymal phenotype by epithelial cells, and it is characterized by loss of tight/adherent junctions (e.g. E‐cadherin, claudins, occludins, and desmosomes) [36] and activation of mesenchymal proteins (e.g. N‐Cadherin, α‐smooth muscle actin, fibronectin, and vimentin) that ultimately lead to enhanced cell migration, invasion, and metastasis [60]. Thus, the evidence that CFTR loss‐of‐function promotes EMT further supports the hypothesis that CFTR may act as a tumor suppressor. However, different *CFTR* mutations may differentially affect the molecular and cellular mechanisms that ultimately lead to CF pathology [61]. In line with this, a recent report demonstrates important distinctions in the expression levels of a few EMT genes and proteins between F508del and KO lung rats and primary rat tracheal basal epithelial cells [58]. Despite the obvious differences in models that make it difficult to compare our proteomic investigation with this study, the key aspect that emerges is that EMT is activated in CF with remarkable differences due to the presence of mutated *CFTR* or its depletion. In addition, a side‐by‐side comparison with human CF bronchial tissues and primary cells from F508del/F508del, R347P/711 + 5 G > A, and M1101K/1609delCA individuals [57] highlights a similar modulation of some EMT markers (OCLN, KRT18, cadherins, and collagens) but not others as compared to our work. Thus, although the overall effect is to increase EMT markers, transcriptional and proteomic pathways may differ among *CFTR* genotypes and in the absence of CFTR expression. Further studies are needed to clarify this point.

CF airways are affected by aberrant inflammation, marked by abundant parenchymal infiltration of PMN, which release a repertoire of tissue‐damaging enzymes, including neutrophilic elastase (NE), as well as high amounts of DNA‐enriched neutrophil extracellular TRAPs (NETs) [62]. This contributes to the progression of CF pulmonary disease, and strategies to limit PMN recruitment, elastase activity, and release of NETs have been proposed as new therapeutic options for the treatment of CF [63, 64, 65]. Consistent with this, the CFTR‐KO cells exhibited a significant increase in the secretion of several pro‐inflammatory cytokines and chemokines (Fig. 4), thereby providing evidence of direct CFTR‐driven regulation of pro‐inflammatory pathways, in agreement with the observation of early lung inflammation in the absence of infection in people with CF [5]. Moreover, the proteomic data revealed a significant upregulation of the NET signaling pathway (Fig. 3), suggesting that in addition to being exposed to enhanced NET release by infiltrating PMN, CF airways may suffer from altered NET‐triggered signaling that can amplify NET‐induced epithelial damage. These aspects, however, require further investigation.

Experiments with the airway on chip provided functional readouts of the invasive and inflammatory phenotype consequent to CFTR ablation. Consistent with *in vivo* evidence [66, 67] and with our previous work on the assembly of a CF airway‐on‐a‐chip [29] populated with F508del/F508del human airway epithelial cells, chips with CFTR‐KO cells exhibited increased PMN recruitment (Fig. 4). This is also consistent with proteomic data showing enhanced chemotaxis of immune cells among the pathways modulated by CFTR‐KO (Fig. 3).

On the contrary, organ chips revealed increased migratory and invasive capability of the CFTR‐KO cells toward the endothelial cell‐lined vascular channel (Fig. 5). In addition, analysis of human data sets linked *CFTR* expression to microtubule changes that are indicative of cell movement and metastasis (Fig. 6). These findings are consistent with the proteomic data showing that CFTR‐KO was associated with the overexpression of EMT‐related proteins (i.e. CD44, PLAUR, and EPHA2), significant modulation of adherent junctions, and the activation of mTOR‐ and Myc‐mediated tumorigenesis pathways (Figs 2 and 3). Thus, CFTR loss‐of‐function significantly impacts multiple cellular pathways, many of which are related to cancer biology. Indeed, the c‐Myc oncogene, mTORC1, and the E2F family of transcription factors are critical regulators of cellular processes, such as growth, metabolism, and the cell cycle [68, 69], and their dysregulation is a common feature in many cancers, leading to uncontrolled proliferation and survival of cancer cells [70].

The downregulation of proteins linked to c‐Myc, mTORC1, and E2F family indicates a potential suppression of these pathways in CFTR‐KO cells. Conversely, the upregulation of proteins involved in the EMT program, a process often associated with cancer metastasis, suggests that the absence of CFTR leads to a shift toward a more aggressive cellular phenotype. This evidence, together with the organ‐on‐chip data showing enhanced epithelial cell invasiveness, adds relevant elements to our understanding of CFTR pathobiology, particularly within the context of tumorigenesis. In addition, the organ‐on‐chip evidence sets the basis for the development of *in vitro* assays for studies on EMT and its pharmacological modulation.

In conclusion, our *in vitro* model with isogenic WT and CFTR‐KO cells provides strong evidence of the multifunctional properties of this protein. We demonstrate the direct impact of CFTR expression and function on inflammation and processes related to carcinogenesis. These results can contribute to a better understanding of CFTR‐dependent signaling pathways in the perspective of uncovering druggable targets for CF as well as other disease states where nongenetic CFTR loss‐of‐function does occur.

## Materials and methods

### Cells

The human bronchial epithelial cell line 16HBE14o‐ (RRID: CVCL_0112) was kindly provided by Dr. Dieter Gruenert, UCSF, USA. WT and CFTR‐KO 16HBE14o‐ were cultured in minimum essential medium (MEM, Invitrogen, Carlsbad, CA, USA) containing 10% fetal bovine serum (FBS), 1% penicillin/streptomycin (P/S) and 1% L‐glutamine. PAEC were cultured as previously described [10, 11], in a custom culture medium composed of 50% vol/vol DMEM‐M199 medium, supplemented with 10% FBS, 1% L‐glutamine, 1% penicillin/streptomycin, 1% endothelial cell growth factor (ECGF), and 1% heparin. PAECs were isolated from pulmonary artery fragments as we previously described [71]. In brief, small pulmonary artery samples were washed with phosphate‐buffered saline (PBS) without calcium and magnesium chloride (PBS^−/−^) and incubated with 2 mg·mL^−1^ type II collagenase (Worthington) in PBS^−/−^ for enzymatic digestion. Subsequently, the digested samples were gently massaged with a spatula (to facilitate cell detachment), rinsed with PBS^−/−^, and the cell‐containing solution was filtered using decreasing mesh size strainers. The cells contained in the eluate were then sedimented by centrifugation, resuspended in culture medium, and expanded.

293‐T cells (ATCC, RRID: CVCL_0063), used for viral particle production, were cultured in DMEM containing 4.5 g·L^−1^ glucose, 10% FBS, 1% P/S, and 1% L‐glutamine. All cell lines have been authenticated in the past 3 years. The typical epithelial cell shape of WT and CFTR‐KO clones was proved by morphological analysis, using phase‐contrast microscopy, and the positivity for the EpCAM epithelial cell marker was assessed by immunocytochemistry. Moreover, western blot analysis was used to confirm the presence of the CFTR protein in WT clones. Phase‐contrast microscopy and immunocytochemistry were also used to verify the typical endothelial shape of PAECs and their positivity to the CD31 endothelial cell marker. All experiments were performed with mycoplasma‐free cells.

### Chip cultures

16HBE14o‐ cells (WT or CFTR‐KO) were co‐cultured with PAEC in two separate channels of the Chip‐S1 devices (Emulate® Inc., Boston, MA, USA). The polydimethylsiloxane chips contain two parallel linear channels separated by a 7 μm pore membrane. Chips were activated using the ER1/ER2 reagents following the manufacturer's instructions, as previously described [29] and coated overnight at 37 °C with an extracellular matrix composed of 50 μg·mL^−1^ human fibronectin, 50 μg·mL^−1^ human laminin, and 100 μg·mL^−1^ type I collagen. The next day, the chips were washed twice with PBS and ~20 μL of primary PAEC suspended at 5 × 10^6^ cells·mL^−1^ in endothelial growth medium were seeded in the bottom channel of the chips and let them adhere by inverting the chips for 1 h at 37 °C. Chips were then inverted back and ~35 μL of WT or CFTR‐KO 16HBE14o‐ cells suspended at 3 × 10^6^ cells·mL^−1^ in epithelial growth medium were seeded in the upper channel of the chip. After 2 h, the channels were rinsed with 200 μL fresh medium and tips were left inserted to prevent drying and allow cell adhesion overnight. The chips were then placed into the Pods^®^ and inserted in the Zoe® module (Emulate^®^ Inc.), as indicated by the manufacturer. Both apical and bottom channels were perfused at a flow rate of 45 μL·h^−1^ for 3 days with the degassed endothelial or epithelial medium. The endothelial growth medium was supplemented with 10 ng·mL^−1^ vascular endothelial growth factor (VEGF, Merck, Darmstadt, Germany) and 1 μg·mL^−1^ ascorbic acid.

PMN were isolated from peripheral blood as previously described [29]. This procedure usually gives 90–95% purity. PMN were suspended at a concentration of 4 × 10^6^/mL, live‐stained using the 5(6)‐Carboxyfluorescein Diacetate N‐Succinimidyl Ester (CFSE) cell tracker (Sigma‐Aldrich, Milan, Italy) and perfused through the bottom channel of the chips for 30 min at 500 μL·h^−1^. Green‐fluorescent PMN adhered to the lower side of the membrane were imaged using a Zeiss LSM800 Confocal Laser Scanning Microscope (Carl Zeiss, Oberkochen, Germany).

### PMN quantification

To quantify PMN recruitment, 10 images were taken for each chip along the entire length of the channel. In total, two control chips and three KO chips were analyzed. The quantification of PMNs was performed using the Fiji software as follows: first, the background was removed from the images (setting: 10 pixels), the images were converted to 8‐bit. A threshold between 25 and 255 was applied, and the ‘fill holes’ and ‘watershed’ settings were used sequentially. The particles were then analyzed using the ‘30‐infinity’ setting.

### CRISPR/Cas9 ablation of CFTR

The sgRNA GCGCCCGAGAGACCATGCAG, targeting exon 1 of CFTR, was inserted into transEDIT pCLIP‐ALL‐hCMV‐Puro plus Cas9 plasmids (Transomic, Huntsville, AL, USA), while the nontargeting control vector contained a scrambled sgRNA sequence (GGAGCGCACCATCTTCTTCA). 293‐T cells were co‐transfected using calcium phosphate, as previously reported [72], with targeting and control plasmids together with GAG‐ and POL‐coding plasmids in order to produce viral particles. The supernatant containing viral particles was collected after 40 h, filtered using 0.45 μm filters, and then used to infect 16HBE14o‐cells. Infected cells were selected using 3 μg·mL^−1^ puromycin and single‐cell seeded on a 96‐well plate to generate clones. Genomic DNA was extracted from the clones using the Genomic DNA Purification Kit (Promega, Madison, WI, USA) and the following primers GAAGTCACCAAAGCAGTACAGC (for) and AAATGGCTGGGTGTAGGAGC (rev) were used for PCR amplification of the region containing the sgRNA targeting sequence. PCR bands were purified and sequenced.

### Western blotting

Cells were lysed at confluency using a buffer (pH 7.5) containing 50 mm Tris, 150 mm NaCl, 0.1% SDS, 1% sodium deoxycholate, and 1% Triton X‐100 supplemented with 2x‐concentrated complete mini protease inhibitor (Roche, Basel, Switzerland). Thirty micrograms of protein lysate were run on an 8% polyacrylamide gel and transferred to a nitrocellulose membrane. The membrane was blocked overnight with 3% milk in TBS‐T (0.1% Tween‐20 in TBS), incubated for 2 h at room temperature (RT) with 596 anti‐CFTR primary antibody (obtained from the University of North Carolina, CF Foundation) 1:1000, or with anti β‐actin (#A5441, Sigma‐Aldrich™) diluted 1:5000 with 3% milk in TBS‐T and incubated for 1 h with goat anti‐mouse secondary antibody (Merck Millipore, Burlington, MA, USA) 1:5000 diluted with 3% milk.

### CFTR activity

CFTR activity in WT and CFTR‐KO clones was evaluated using a live microscopy assay based on the yellow fluorescent protein (YFP) mutant YFP‐H148Q, which is a sensor of pH and halides [30]. In brief, cells were seeded in replicates in a 24‐well imaging plate (Eppendorf, Milan, Italy) transfected with the modified YFP‐H148Q‐pCDNA3 plasmid (kindly provided by Dr. Luis Galietta, Telethon Institute of Genetics and Medicine TIGEM, Pozzuoli, Italy) using Lipofectamine‐2000 (Invitrogen). Forty‐eight hours after transfection, the fluorescence decay after the addition of 137 mm sodium iodide was monitored using the ‘Axio Cam’ fluorescence microscope (Axio‐vision, Carl Zeiss, Oberkochen, Germany).

### Proteomics

For proteomics, 3 WT‐ and 3 CFTR‐KO 16HBE14o‐ clones were lysed with a buffer (6 m urea in 100 mm Tris/HCl, pH 7.5) by sonication. After the removal of cell debris by centrifugation for 30 min at 14 000 ***g***, 4 °C, supernatants were used for protein quantification via Bradford assay (Bio‐Rad, Hercules, CA, USA) using Bovine Serum Albumin (BSA, Sigma‐Aldrich™) standard for the calibration curve to digest 30 μg of proteins for WT and KO clone samples. Filter‐aided sample preparation (FASP) tryptic digestion was carried out overnight at 37 °C using trypsin (Sigma‐Aldrich™). As previously described [73, 74], tryptic peptides from each sample were analyzed in triplicate by nanoLC–MS/MS using the UltiMate™ 3000 UPLC (Thermo Fisher Scientific, Waltham, MA, USA) chromatographic system coupled to the Orbitrap Fusion™ Tribrid™ (Thermo Fisher Scientific) mass spectrometer.

After LC–MS/MS analyses, raw files were processed using free computational proteomics platforms, MaxQuant version 1.6.6.0 and Perseus version 1.6.10.50 (Max‐Planck Institute for Biochemistry, Martinsried, Germany). Peak lists were searched using the Andromeda [75] peptide search engine against the UniProt database (released 2020_06, taxonomy Homo Sapiens, 20 588 entries). Processing parameters and details are listed in published work [74, 76]. Briefly, LFQ Intensities were used for calculating the protein abundance ratio (CFTR‐KO/WT) and for performing functional analysis through the IPA (Qiagen, Hilden, Germany) bioinformatics tool to evaluate and predict biological functions potentially modulated and upstream regulators related to the acquired protein dataset.

### Cytokine and chemokine release

For cytokine experiments, 5 × 10^4^ cells·well^−1^ CFTR‐KO and WT 16HBE14o‐cells were seeded in 96‐well plates in EMEM supplemented with 2% FBS, and cell supernatants were harvested after 24 h of culture. IL‐8 and IL‐6 quantification in the supernatants of the cell culture was performed by ELISA (ELISA ReadysetGo, Invitrogen).

A panel of 14 chemo‐cytokines was analyzed by multiplex immunoassay using the Luminex technology. Cell supernatants were analyzed using Human Custom Invitrogen™ ProcartaPlex™, 14‐plex (Invitrogen), which included 14 different analytes: eotaxin (CCL11), IFN‐γ, IL‐1α, IL‐1β, IL12p40, IL‐3, IL‐6, IL‐7, IL‐8 (CXCL8), IP‐10 (CXCL10), MCP‐1 (CCL2), RANTES (CCL5), TNF‐α, and VEGF‐A. Completed assays were read on a Bio‐Plex 200 system (Bio‐Rad).

### Immunocytochemistry

Cells on the chips were fixed by incubation with 4% paraformaldehyde (PFA) in PBS for 30 min. Subsequently, cells were permeabilized by incubation for 1 h with a 0.2% Triton X‐100 solution in PBS and blocked using a solution containing 5% goat serum and 0.1% Triton X‐100 in PBS. The same solution was used for antibody incubations. For the visualization of endothelial cells, an anti‐CD31 antibody (M0823, Dako, 1:50 dilution, 1‐h incubation at RT produced in mouse was used). Cells were then washed three times with PBS and incubated with an AlexaFluor goat anti‐mouse antibody (Invitrogen) diluted 1:300. Subsequently, cells were incubated for 1 h at RT with an antibody (house made in Trerotola's Lab) directed against the EpCAM protein and conjugated to 633 (house made in Trerotola's Lab). Nuclei were stained by incubating with 1 μg·mL^−1^ DAPI (Sigma‐Aldrich) for 10 min.

### Quantification of the endothelial damage

The endothelial cells on the lower side of the chip membrane, labeled with the anti‐CD31 fluorescent antibody, were imaged by tile imaging with a LSM800 confocal microscope (Zeiss). A median filter was applied to the images to remove speckle noises from the background, using imagej. The processed images were then analyzed by the ‘CellProfiler’ [77] for morphological feature extraction. The images were first cropped to exclude the curving regions of the microfluidic channel and then inverted to better segment individual cells for images without nuclear labeling. The cells were segmented based on CD31 staining with ‘Global’ threshold strategy and the ‘Otsu’ method of ‘IdentifyPrimaryObjects’ CellProfiler module. The resulting objects were further filtered based on the object intensity to reduce false segmentation. Cell morphology and neighboring information were measured and exported for statistical analysis. The relative sum area occupied by ECs was quantified and used as the metric for assessing the integrity of the endothelial layer.

### MSigDB, DAVID and IPA analysis

The molecular signature database (MSigDB v7.0; UC San Diego, CA, USA, and Broad Institute, Boston, MA, USA) [78] was interrogated to compute overlapping analysis of CFTR‐KO regulated genes, in proteomic screening, with the Hallmark gene sets (*N* = 50), which represent ‘specific well‐defined biological states or processes and display coherent expression’ [78].

For the functional annotation and analysis, the Database for Annotation, Visualization and Integrated Discovery (DAVID) v6.8 was used. Protein identifiers from UniProtKB were used as input in DAVID, and specific annotation categories, such as ‘Biological Process’ to categorize the proteins were selected.

IPA was used to identify target genes, biological functions, and diseases modulated by CFTR deletion. Differentially expressed proteins among WT and CFTR‐KO cells (defined as q‐value <0.05 and log_2_ fold change = 1) were loaded on IPA to detect the activation states (increased or decreased) of biological pathways modulated in the CFTR‐KO cells.

### Growth assay

For the impedance‐based real‐time cell analysis, 3 × 10^4^ CFTR‐WT or CFTR‐KO cells were seeded on iCelligence E‐plate L8 (ACEA Biosciences, San Diego, CA, USA) into independent wells (in duplicates), inserted into the ACEA iCELLigence instrument, and cultured at 37 °C, 5% CO_2_. Cell growth was monitored continuously up to 45 h and analyzed using the RTCA analysis software, as we previously reported [79].

### TCGA dataset analysis

Survival analysis, differential gene expression, and GO were analyzed from RNA‐seq and clinical information of LUAD patients collected within the TGCA project as we previously reported [80]. Briefly, RNA‐seq and clinical data were collected from cBioPortal (cbioportal.org/datasets, accessed on 2 August 2024). Patients with available RNA‐seq data were stratified in low *CFTR* (4th quartile of *CFTR* expression, *n* = 127) and high *CFTR* (1st quartile). Survival analysis and differentially expressed genes (DEG) among these two groups were retrieved from cBioportal. DEG were identified as −1 > log_2_FC >1 and false discovery rate (FDR) <0.05. Gene Ontology of DEG was analyzed with SRplot to evidence enrichment in cellular components, molecular functions, and biological processes of the identified gene set.

### Human samples

The study methodologies conformed to the standards set by the Declaration of Helsinki. The isolation of PAEC from human specimens, collected in 2019 at the Polyclinic Hospital of Chieti, followed the guidelines of the human research ethics committee of the University of Chieti‐Pescara (#237_2018bis). PMN, collected in 2023 at the Center for Advanced Studies and Technology, University of Chieti‐Pescara, were isolated from blood drawn from healthy volunteers recruited in the laboratory who signed an informed consent form.

### Statistical analysis

Statistical analyses were performed using GraphPad Prism (GraphPad, San Diego, CA, USA). Results are shown as mean ± standard deviation (SD) or standard error of the mean (SEM), as indicated. Cytokine measurements were analyzed with an unpaired *t*‐test per row, with individual variances computed for each comparison and adjusted with the Bonferroni‐Dunn's multiple‐comparison test. The CFTR activity was evaluated through one experiment with 6 replicates per each condition. The mean fluorescence intensity was calculated per time point in each replicate. Data points represent mean of the six replicates ± SEM and were analyzed using two‐way ANOVA and Sidak's multiple‐comparison test. For the quantification of PMN recruitment, 10 field images were taken by confocal microscopy on the basal side of the membrane, along the entire length of the channel in each chip (*n* = 2 WT chips and *n* = 3 KO chips from one experiment). The cell number of each image was evaluated, and all measured values (20 for the WT chips and 30 for the KO chips) were used to estimate the relative PMN recruitment. The results were analyzed using the GraphPad Prism™ software. Results are presented as mean ± SEM, and nonparametric data distribution was assessed using the Mann–Whitney T‐test. GSEA calculates four statistics for the gene set enrichment analysis report: (i) Enrichment Score (ES), (ii) Normalized Enrichment Score (NES), (iii) FDR and (iv) Nominal p‐value. The activation z‐scores and statistical significance (*P*‐value) of functions associated with the DEPs were calculated using the IPA core analysis pathways with a z‐score > 2 and *q*‐value <0.05 considered significant. Imaging features generated from CellProfiler were analyzed for statistical significance using the Student's T‐test.

## Conflict of interest

H.B. and Y.C.Y. are employees of Xellar Inc., which specializes in combining human organ chip models with imaging for disease modeling and drug testing. It should be noted that the devices employed in the research presented in this manuscript are not sourced from Xellar Inc.

## Author contributions

RP and DM designed the study and generated the WT and KO clones. MCC, DP, and PDB generated the proteomic data. DM, HB, and TC analyzed and interpreted the proteomic data. LB ran chip experiments. RP, SC, and MMU validated the WT and KO clones. MMA, VL, and CS ran cytokine quantification and analyzed the data. YCY and HB ran the analysis with CellProfiler. MTRED and SDO ran the growth curves and analyzed the data. MTRED designed the graphical abstract. DM, HB, LS, MZ, MTRER, TC, VL, CS, MR, and RP drafted the manuscript and interpreted the data. RP and MR mentored the study. All the authors contributed to providing feedback and writing the manuscript, approved the final version of the manuscript, and agreed to be accountable for all aspects of the work.

## Peer review

The peer review history for this article is available at https://www.webofscience.com/api/gateway/wos/peer‐review/10.1111/febs.70050.

## Supporting information


**Table S1.** Complete list of identified proteins and comparison between CFTR‐KO vs. WT cells.

**Table S2.** MsigDB results on DEPs in CFTR‐KO vs. WT cells.

**Table S3.** Complete list of downregulated proteins ‘Proteins in Overlap (k)’ in CFTR‐KO vs. WT cells (MsigDB results).

**Table S4.** Complete list of upregulated proteins ‘Proteins in Overlap (k)’ in CFTR‐KO vs. WT cells (MsigDB results).

**Table S5.** Analysis of differential expressed proteins using UniProt Key Words (UP KW) linked to Biological Processes in the DAVID database.

## Data Availability

The data supporting the proteomic analysis are available in Table S1. Additional data can be provided by the corresponding authors at roberto.plebani@unich.it or mromano@unich.it upon reasonable request.

## References

[1] Riordan JR , Rommens JM , Kerem B , Alon N , Rozmahel R , Grzelczak Z , Zielenski J , Lok S , Plavsic N , Chou JL *et al*. (1989) Identification of the cystic fibrosis gene: cloning and characterization of complementary DNA. Science 245, 1066–1073. 2475911 10.1126/science.2475911

[2] Saint‐Criq V & Gray MA (2017) Role of CFTR in epithelial physiology. Cell Mol Life Sci 74, 93–115. 27714410 10.1007/s00018-016-2391-y PMC5209439

[3] Stoltz DA , Meyerholz DK & Welsh MJ (2015) Origins of cystic fibrosis lung disease. N Engl J Med 372, 351–362. 25607428 10.1056/NEJMra1300109 PMC4916857

[4] Cantin AM , Hartl D , Konstan MW & Chmiel JF (2015) Inflammation in cystic fibrosis lung disease: pathogenesis and therapy. J Cyst Fibros 14, 419–430. 25814049 10.1016/j.jcf.2015.03.003

[5] Balázs A & Mall MA (2019) Mucus obstruction and inflammation in early cystic fibrosis lung disease: emerging role of the IL‐1 signaling pathway. Pediatr Pulmonol 54(Suppl 3), S5–S12. 10.1002/ppul.24462 31715090

[6] Madácsy T , Pallagi P & Maleth J (2018) Cystic fibrosis of the pancreas: the role of CFTR Channel in the regulation of intracellular Ca^2+^ signaling and mitochondrial function in the exocrine pancreas. Front Physiol 9, 1585. 30618777 10.3389/fphys.2018.01585 PMC6306458

[7] Kinnman N , Lindblad A , Housset C , Buentke E , Scheynius A , Strandvik B & Hultcrantz R (2000) Expression of cystic fibrosis transmembrane conductance regulator in liver tissue from patients with cystic fibrosis. Hepatology 32, 334–340. 10915740 10.1053/jhep.2000.9111

[8] De Lisle RC & Borowitz D (2013) The cystic fibrosis intestine. Cold Spring Harb Perspect Med 3, a009753. 23788646 10.1101/cshperspect.a009753 PMC3753720

[9] Tousson A , Van Tine BA , Naren AP , Shaw GM & Schwiebert LM (1998) Characterization of CFTR expression and chloride channel activity in human endothelia. American Journal of Physiology‐Cell Physiology 275, C1555–C1564. 10.1152/ajpcell.1998.275.6.C1555 9843717

[10] Totani L , Plebani R , Piccoli A , Di Silvestre S , Lanuti P , Recchiuti A , Cianci E , Dell'Elba G , Sacchetti S , Patruno S *et al*. (2017) Mechanisms of endothelial cell dysfunction in cystic fibrosis. Biochim Biophys Acta Mol Basis Dis 1863, 3243–3253. 28847515 10.1016/j.bbadis.2017.08.011

[11] Plebani R , Tripaldi R , Lanuti P , Recchiuti A , Patruno S , Di Silvestre S , Simeone P , Anile M , Venuta F , Prioletta M *et al*. (2017) Establishment and long‐term culture of human cystic fibrosis endothelial cells. Lab Invest 97, 1375–1384. 28759010 10.1038/labinvest.2017.74

[12] Porto PD , Cifani N , Guarnieri S , Di Domenico EG , Mariggiò MA , Spadaro F , Guglietta S , Anile M , Venuta F , Quattrucci S *et al*. (2011) Dysfunctional CFTR alters the bactericidal activity of human macrophages against *Pseudomonas aeruginosa* . PLoS One 6, e19970. 21625641 10.1371/journal.pone.0019970 PMC3097223

[13] Sorio C , Buffelli M , Angiari C , Ettorre M , Johansson J , Vezzalini M , Viviani L , Ricciardi M , Verzè G , Assael BM *et al*. (2011) Defective CFTR expression and function are detectable in blood monocytes: development of a new blood test for cystic fibrosis. PLoS One 6, e22212. 21811577 10.1371/journal.pone.0022212 PMC3141019

[14] Mattoscio D , Evangelista V , De Cristofaro R , Recchiuti A , Pandolfi A , Di Silvestre S , Manarini S , Martelli N , Rocca B , Petrucci G *et al*. (2010) Cystic fibrosis transmembrane conductance regulator (CFTR) expression in human platelets: impact on mediators and mechanisms of the inflammatory response. FASEB J 24, 3970–3980. 20530751 10.1096/fj.10-159921

[15] Ortiz‐Muñoz G , Yu MA , Lefrançais E , Mallavia B , Valet C , Tian JJ , Ranucci S , Wang KM , Liu Z , Kwaan N *et al*. (2020) Cystic fibrosis transmembrane conductance regulator dysfunction in platelets drives lung hyperinflammation. J Clin Investig 130, 2041–2053. 31961827 10.1172/JCI129635 PMC7108932

[16] Rout‐Pitt N , Farrow N , Parsons D & Donnelley M (2018) Epithelial mesenchymal transition (EMT): a universal process in lung diseases with implications for cystic fibrosis pathophysiology. Respir Res 19, 136. 30021582 10.1186/s12931-018-0834-8 PMC6052671

[17] Zhang JT , Jiang XH , Xie C , Cheng H , Da Dong J , Wang Y , Fok KL , Zhang XH , Sun TT , Tsang LL *et al*. (2013) Downregulation of CFTR promotes epithelial‐to‐mesenchymal transition and is associated with poor prognosis of breast cancer. Molec Cell Res 1833, 2961–2969. 10.1016/j.bbamcr.2013.07.021 23916755

[18] Neglia JP , FitzSimmons SC , Maisonneuve P , Schöni MH , Schöni‐Affolter F , Corey M & Lowenfels AB (1995) The risk of cancer among patients with cystic fibrosis. Cystic fibrosis and cancer study group. N Engl J Med 332, 494–499. 7830730 10.1056/NEJM199502233320803

[19] Appelt D , Fuchs T , Steinkamp G & Ellemunter H (2022) Malignancies in patients with cystic fibrosis: a case series. J Med Case Reports 16, 27. 10.1186/s13256-021-03234-1 PMC8767710 35042562

[20] Maisonneuve P , Marshall BC , Knapp EA & Lowenfels AB (2013) Cancer risk in cystic fibrosis: a 20‐year nationwide study from the United States. J Natl Cancer Inst 105, 122–129. 23178438 10.1093/jnci/djs481

[21] Hou Y , Guan X , Yang Z & Li C (2016) Emerging role of cystic fibrosis transmembrane conductance regulator – an epithelial chloride channel in gastrointestinal cancers. World J Gastrointest Oncol 8, 282–288. 26989463 10.4251/wjgo.v8.i3.282 PMC4789613

[22] Anderson KJ , Cormier RT & Scott PM (2019) Role of ion channels in gastrointestinal cancer. World J Gastroenterol 25, 5732–5772. 31636470 10.3748/wjg.v25.i38.5732 PMC6801186

[23] Son JW , Kim YJ , Cho HM , Lee SY , Lee SM , Kang J‐K , Lee JU , Lee YM , Kwon SJ , Choi E *et al*. (2011) Promoter hypermethylation of the CFTR gene and clinical/pathological features associated with non‐small cell lung cancer. Respirology 16, 1203–1209. 21585618 10.1111/j.1440-1843.2011.01994.x

[24] Li J , Zhang JT , Jiang X , Shi X , Shen J , Feng F , Chen J , Liu G , He P , Jiang J *et al*. (2015) The cystic fibrosis transmembrane conductance regulator as a biomarker in non‐small cell lung cancer. Int J Oncol 46, 2107–2115. 25760446 10.3892/ijo.2015.2921

[25] Zhang J , Wang Y , Jiang X & Chan HC (2018) Cystic fibrosis transmembrane conductance regulator‐emerging regulator of cancer. Cell Mol Life Sci 75, 1737–1756. 29411041 10.1007/s00018-018-2755-6 PMC11105598

[26] Si L , Bai H , Rodas M , Cao W , Oh CY , Jiang A , Moller R , Hoagland D , Oishi K , Horiuchi S *et al*. (2021) A human‐airway‐on‐a‐chip for the rapid identification of candidate antiviral therapeutics and prophylactics. Nat Biomed Eng 5, 815–829. 33941899 10.1038/s41551-021-00718-9 PMC8387338

[27] Bai H , Si L , Jiang A , Belgur C , Zhai Y , Plebani R , Oh CY , Rodas M , Patil A , Nurani A *et al*. (2022) Mechanical control of innate immune responses against viral infection revealed in a human lung alveolus chip. Nat Commun 13, 1928. 35396513 10.1038/s41467-022-29562-4 PMC8993817

[28] Benam KH , Villenave R , Lucchesi C , Varone A , Hubeau C , Lee H‐H , Alves SE , Salmon M , Ferrante TC , Weaver JC *et al*. (2016) Small airway‐on‐a‐chip enables analysis of human lung inflammation and drug responses in vitro. Nat Methods 13, 151–157. 26689262 10.1038/nmeth.3697

[29] Plebani R , Potla R , Soong M , Bai H , Izadifar Z , Jiang A , Travis RN , Belgur C , Dinis A , Cartwright MJ *et al*. (2021) Modeling pulmonary cystic fibrosis in a human lung airway‐on‐a‐chip. J Cyst Fibros 21, 606–615. 34799298 10.1016/j.jcf.2021.10.004

[30] Galietta LV , Jayaraman S & Verkman AS (2001) Cell‐based assay for high‐throughput quantitative screening of CFTR chloride transport agonists. Am J Physiol Cell Physiol 281, C1734–C1742. 11600438 10.1152/ajpcell.2001.281.5.C1734

[31] Chen C , Zhao S , Karnad A & Freeman JW (2018) The biology and role of CD44 in cancer progression: therapeutic implications. J Hematol Oncol 11, 64. 29747682 10.1186/s13045-018-0605-5 PMC5946470

[32] Wang Q , Wang Y , Zhang Y , Zhang Y & Xiao W (2013) The role of uPAR in epithelial‐mesenchymal transition in small airway epithelium of patients with chronic obstructive pulmonary disease. Respir Res 14, 67. 23806081 10.1186/1465-9921-14-67 PMC3700841

[33] Huang J , Xiao D , Li G , Ma J , Chen P , Yuan W , Hou F , Ge J , Zhong M , Tang Y *et al*. (2014) EphA2 promotes epithelial‐mesenchymal transition through the Wnt/β‐catenin pathway in gastric cancer cells. Oncogene 33, 2737–2747. 23752181 10.1038/onc.2013.238

[34] Pilewski JM & Frizzell RA (1999) Role of CFTR in airway disease. Physiol Rev 79, S215–S255. 9922383 10.1152/physrev.1999.79.1.S215

[35] Lotti V , Merigo F , Lagni A , Di Clemente A , Ligozzi M , Bernardi P , Rossini G , Concia E , Plebani R , Romano M *et al*. (2022) CFTR modulation reduces SARS‐CoV‐2 infection in human bronchial epithelial cells. Cells 11, 1347. 35456026 10.3390/cells11081347 PMC9028056

[36] Greenburg G & Hay ED (1986) Cytodifferentiation and tissue phenotype change during transformation of embryonic lens epithelium to mesenchyme‐like cells in vitro. Dev Biol 115, 363–379. 3519318 10.1016/0012-1606(86)90256-3

[37] Nichols DP & Chmiel JF (2015) Inflammation and its genesis in cystic fibrosis. Pediatr Pulmonol 50, S39–S56. 26335954 10.1002/ppul.23242

[38] Mani SA , Guo W , Liao M‐J , Eaton EN , Ayyanan A , Zhou AY , Brooks M , Reinhard F , Zhang CC , Shipitsin M *et al*. (2008) The epithelial‐mesenchymal transition generates cells with properties of stem cells. Cell 133, 704–715. 18485877 10.1016/j.cell.2008.03.027 PMC2728032

[39] Evdokimova V , Tognon C , Ng T & Sorensen PHB (2009) Reduced proliferation and enhanced migration: two sides of the same coin? Molecular mechanisms of metastatic progression by YB‐1. Cell Cycle 8, 2901–2906. 19713745 10.4161/cc.8.18.9537

[40] Koh YW , Han J‐H & Haam S (2021) Expression of PD‐L1, cancer stem cell and epithelial‐mesenchymal transition phenotype in non‐small cell lung cancer. Pathology 53, 239–246. 33036771 10.1016/j.pathol.2020.07.009

[41] Shi X , Kou M , Dong X , Zhai J , Liu X , Lu D , Ni Z , Jiang J & Cai K (2022) Integrative pan cancer analysis reveals the importance of CFTR in lung adenocarcinoma prognosis. Genomics 114, 110279. 35134493 10.1016/j.ygeno.2022.110279

[42] Xiao Q , Koutsilieri S , Sismanoglou D‐C & Lauschke VM (2022) CFTR reduces the proliferation of lung adenocarcinoma and is a strong predictor of survival in both smokers and non‐smokers. J Cancer Res Clin Oncol 148, 3293–3302. 35715537 10.1007/s00432-022-04106-x PMC9587080

[43] Whipple RA , Matrone MA , Cho EH , Balzer EM , Vitolo MI , Yoon JR , Ioffe OB , Tuttle KC , Yang J & Martin SS (2010) Epithelial‐to‐mesenchymal transition promotes tubulin detyrosination and microtentacles that enhance endothelial engagement. Cancer Res 70, 8127–8137. 20924103 10.1158/0008-5472.CAN-09-4613 PMC3123454

[44] Pezzulo AA , Starner TD , Scheetz TE , Traver GL , Tilley AE , Harvey B‐G , Crystal RG , McCray PB & Zabner J (2011) The air‐liquid interface and use of primary cell cultures are important to recapitulate the transcriptional profile of in vivo airway epithelia. Am J Physiol 300, L25–L31. 10.1152/ajplung.00256.2010 PMC3023285 20971803

[45] Haws C , Krouse ME , Xia Y , Gruenert DC & Wine JJ (1992) CFTR channels in immortalized human airway cells. Am J Physiol 263, L692–L707. 1282304 10.1152/ajplung.1992.263.6.L692

[46] Forbes B , Shah A , Martin GP & Lansley AB (2003) The human bronchial epithelial cell line 16HBE14o − as a model system of the airways for studying drug transport. Int J Pharm 257, 161–167. 12711171 10.1016/s0378-5173(03)00129-7

[47] Cozens AL , Yezzi MJ , Kunzelmann K , Ohrui T , Chin L , Eng K , Finkbeiner WE , Widdicombe JH & Gruenert DC (1994) CFTR expression and chloride secretion in polarized immortal human bronchial epithelial cells. Am J Respir Cell Mol Biol 10, 38–47. 7507342 10.1165/ajrcmb.10.1.7507342

[48] Callaghan PJ , Ferrick B , Rybakovsky E , Thomas S & Mullin JM (2020) Epithelial barrier function properties of the 16HBE14o – human bronchial epithelial cell culture model. Biosci Rep 40, BSR20201532. 32985670 10.1042/BSR20201532 PMC7569203

[49] Van den Bossche S , Ostyn L , Vandendriessche V , Rigauts C , De Keersmaecker H , Nickerson CA & Crabbé A (2023) The development and characterization of *in vivo*‐like three‐dimensional models of bronchial epithelial cell lines. Eur J Pharm Sci 190, 106567. 37633341 10.1016/j.ejps.2023.106567

[50] Hackett T‐L , Warner SM , Stefanowicz D , Shaheen F , Pechkovsky DV , Murray LA , Argentieri R , Kicic A , Stick SM , Bai TR *et al*. (2009) Induction of epithelial–mesenchymal transition in primary airway epithelial cells from patients with asthma by transforming growth factor‐β1. Am J Respir Crit Care Med 180, 122–133. 19406982 10.1164/rccm.200811-1730OC

[51] Pankow S , Bamberger C , Calzolari D , Martínez‐Bartolomé S , Lavallée‐Adam M , Balch WE & Yates JR (2015) ∆F508 CFTR interactome remodelling promotes rescue of cystic fibrosis. Nature 528, 510–516. 26618866 10.1038/nature15729 PMC4826614

[52] Braccia C , Tomati V , Caci E , Pedemonte N & Armirotti A (2019) SWATH label‐free proteomics for cystic fibrosis research. J Cyst Fibros 18, 501–506. 30348611 10.1016/j.jcf.2018.10.004

[53] Birch RJ , Peckham D , Wood HM , Quirke P , Konstant‐Hambling R , Brownlee K , Cosgriff R , Consortium GER , Burr N & Downing A (2023) The risk of colorectal cancer in individuals with mutations of the cystic fibrosis transmembrane conductance regulator (CFTR) gene: an English population‐based study. J Cyst Fibros 22, 499–504. 36253274 10.1016/j.jcf.2022.10.001

[54] Tu Z , Chen Q , Zhang JT , Jiang X , Xia Y & Chan HC (2016) CFTR is a potential marker for nasopharyngeal carcinoma prognosis and metastasis. Oncotarget 7, 76955–76965. 27769067 10.18632/oncotarget.12762 PMC5363562

[55] Kamal S , Babar S , Ali W , Rehman K , Hussain A & Akash MSH (2024) Sirtuin insights: bridging the gap between cellular processes and therapeutic applications. Naunyn Schmiedebergs Arch Pharmacol 397, 9315–9344. 38976046 10.1007/s00210-024-03263-9

[56] Sun Q , Lin X , Zhao Y , Li L , Yan K , Liang D , Sun D & Li Z‐C (2020) Deep learning vs. radiomics for predicting axillary lymph node metastasis of breast cancer using ultrasound images: Don't forget the peritumoral region. Front Oncol 10, 53. 32083007 10.3389/fonc.2020.00053 PMC7006026

[57] Quaresma MC , Pankonien I , Clarke LA , Sousa LS , Silva IAL , Railean V , Doušová T , Fuxe J & Amaral MD (2020) Mutant CFTR drives TWIST1 mediated epithelial‐mesenchymal transition. Cell Death Dis 11, 920. 33106471 10.1038/s41419-020-03119-z PMC7588414

[58] Rout‐Pitt N , Boog B , McCarron A , Reyne N , Parsons D & Donnelley M (2024) Insights into epithelial‐mesenchymal transition from cystic fibrosis rat models. J Cyst Fibros 24, 149–156. 39266334 10.1016/j.jcf.2024.09.003

[59] Sun TT , Wang Y , Cheng H , Xiao HZ , Xiang JJ , Zhang JT , Yu SBS , Martin TA , Ye L , Tsang LL *et al*. (2014) Disrupted interaction between CFTR and AF‐6/afadin aggravates malignant phenotypes of colon cancer. Biochim Biophys Acta 1843, 618–628. 24373847 10.1016/j.bbamcr.2013.12.013

[60] Jonsdottir HR , Arason AJ , Palsson R , Franzdottir SR , Gudbjartsson T , Isaksson HJ , Gudmundsson G , Gudjonsson T & Magnusson MK (2015) Basal cells of the human airways acquire mesenchymal traits in idiopathic pulmonary fibrosis and in culture. Lab Invest 95, 1418–1428. 26390052 10.1038/labinvest.2015.114

[61] Santos L , Nascimento R , Duarte A , Railean V , Amaral MD , Harrison PT , Gama‐Carvalho M & Farinha CM (2023) Mutation‐class dependent signatures outweigh disease‐associated processes in cystic fibrosis cells. Cell Biosci 13, 26. 36759923 10.1186/s13578-023-00975-y PMC9912517

[62] Gifford AM & Chalmers JD (2014) The role of neutrophils in cystic fibrosis. Curr Opin Hematol 21, 16–22. 24253427 10.1097/MOH.0000000000000009

[63] Recchiuti A , Patruno S , Plebani R & Romano M (2020) The resolution approach to cystic fibrosis inflammation. Front Pharmacol 11, 1129. 32848748 10.3389/fphar.2020.01129 PMC7403222

[64] Totani L , Amore C , Piccoli A , Dell'Elba G , Di Santo A , Plebani R , Pecce R , Martelli N , Rossi A , Ranucci S *et al*. (2021) Type‐4 phosphodiesterase (PDE4) blockade reduces NETosis in cystic fibrosis. Front Pharmacol 12, 702677. 34566635 10.3389/fphar.2021.702677 PMC8456009

[65] Tucker SL , Sarr D & Rada B (2021) Neutrophil extracellular traps are present in the airways of ENaC‐overexpressing mice with cystic fibrosis‐like lung disease. BMC Immunol 22, 7. 33478382 10.1186/s12865-021-00397-w PMC7819174

[66] Hubeau C , Lorenzato M , Couetil JP , Hubert D , Dusser D , Puchelle E & Gaillard D (2001) Quantitative analysis of inflammatory cells infiltrating the cystic fibrosis airway mucosa. Clin Exp Immunol 124, 69–76. 11359444 10.1046/j.1365-2249.2001.01456.x PMC1906034

[67] Kirchner KK , Wagener JS , Khan TZ , Copenhaver SC & Accurso FJ (1996) Increased DNA levels in bronchoalveolar lavage fluid obtained from infants with cystic fibrosis. Am J Respir Crit Care Med 154, 1426–1429. 8912759 10.1164/ajrccm.154.5.8912759

[68] Dhanasekaran R , Deutzmann A , Mahauad‐Fernandez WD , Hansen AS , Gouw AM & Felsher DW (2022) The MYC oncogene – the grand orchestrator of cancer growth and immune evasion. Nat Rev Clin Oncol 19, 23–36. 34508258 10.1038/s41571-021-00549-2 PMC9083341

[69] Real S , Meo‐Evoli N , Espada L & Tauler A (2011) E2F1 regulates cellular growth by mTORC1 signaling. PLoS One 6, e16163. 21283628 10.1371/journal.pone.0016163 PMC3026008

[70] Hsieh AL , Walton ZE , Altman BJ , Stine ZE & Dang CV (2015) MYC and metabolism on the path to cancer. Semin Cell Dev Biol 43, 11–21. 26277543 10.1016/j.semcdb.2015.08.003 PMC4818970

[71] Plebani R , D'Alessandro A , Lanuti P , Simeone P , Cinalli M , Palleschi A , Mucci M , Marchisio M , Cappabianca F , Camera M *et al*. Microvascular and macrovascular endothelial cell isolation and purification from lung‐derived samples. J Vis Exp doi: 10.3791/64885 36808144

[72] Mattoscio D , Casadio C , Miccolo C , Maffini F , Raimondi A , Tacchetti C , Gheit T , Tagliabue M , Galimberti VE , De Lorenzi F *et al*. (2017) Autophagy regulates UBC9 levels during viral‐mediated tumorigenesis. PLoS Pathog 13, e1006262. 28253371 10.1371/journal.ppat.1006262 PMC5349695

[73] Cela I , Cufaro MC , Fucito M , Pieragostino D , Lanuti P , Sallese M , Del Boccio P , Di Matteo A , Allocati N , De Laurenzi V *et al*. (2022) Proteomic investigation of the role of nucleostemin in Nucleophosmin‐mutated OCI‐AML 3 cell line. Int J Mol Sci 23, 7655. 35886999 10.3390/ijms23147655 PMC9317519

[74] Potenza F , Cufaro MC , Di Biase L , Panella V , Di Campli A , Ruggieri AG , Dufrusine B , Restelli E , Pietrangelo L , Protasi F *et al*. (2021) Proteomic analysis of Marinesco‐Sjogren syndrome fibroblasts indicates pro‐survival metabolic adaptation to SIL1 loss. Int J Mol Sci 22, 12449. 34830330 10.3390/ijms222212449 PMC8620507

[75] Cox J , Neuhauser N , Michalski A , Scheltema RA , Olsen JV & Mann M (2011) Andromeda: a peptide search engine integrated into the MaxQuant environment. J Proteome Res 10, 1794–1805. 21254760 10.1021/pr101065j

[76] Damiani V , Cufaro MC , Fucito M , Dufrusine B , Rossi C , Del Boccio P , Federici L , Turco MC , Sallese M , Pieragostino D *et al*. (2022) Proteomics approach highlights early changes in human fibroblasts‐pancreatic ductal adenocarcinoma cells crosstalk. Cells 11, 1160. 35406724 10.3390/cells11071160 PMC8997741

[77] Stirling DR , Swain‐Bowden MJ , Lucas AM , Carpenter AE , Cimini BA & Goodman A (2021) CellProfiler 4: improvements in speed, utility and usability. BMC Bioinformatics 22, 433. 34507520 10.1186/s12859-021-04344-9 PMC8431850

[78] Liberzon A , Birger C , Thorvaldsdóttir H , Ghandi M , Mesirov JP & Tamayo P (2015) The molecular signatures database (MSigDB) hallmark gene set collection. Cell Syst 1, 417–425. 26771021 10.1016/j.cels.2015.12.004 PMC4707969

[79] Mattoscio D , Isopi E , Lamolinara A , Patruno S , Medda A , De Cecco F , Chiocca S , Iezzi M , Romano M & Recchiuti A (2021) Resolvin D1 reduces cancer growth stimulating a protective neutrophil‐dependent recruitment of anti‐tumor monocytes. J Exp Clin Cancer Res 40, 129. 33845864 10.1186/s13046-021-01937-3 PMC8040222

[80] Mattoscio D , Ferri G , Miccolo C , Chiocca S , Romano M & Recchiuti A (2022) Gene expression of the D‐series Resolvin pathway predicts activation of anti‐tumor immunity and clinical outcomes in head and neck cancer. IJMS 23, 6473. 35742918 10.3390/ijms23126473 PMC9223893

